# Evolution of External Health Costs of Electricity Generation in the Baltic States

**DOI:** 10.3390/ijerph17155265

**Published:** 2020-07-22

**Authors:** Jintao Lu, Chong Zhang, Licheng Ren, Mengshang Liang, Wadim Strielkowski, Justas Streimikis

**Affiliations:** 1 Department of Business Administration, School of Economics and Management, Taiyuan University of Science and Technology, Taiyuan 030024, China; zhangchong@tyust.edu.cn (C.Z.); s20180652@stu.tyust.edu.cn (L.R.); S20190749@stu.tyust.edu.cn (M.L.); 2 Research Center for Corporate Social Responsibility, Taiyuan University of Science and Technology, Taiyuan 030024, China; 3 Department of Trade and Finance, Faculty of Economics and Management, Czech University of Life Sciences Prague, Kamýcká 129, 16500 Prague, Czech Republic; 4 Division of Farms and Enterprises Economics, Lithuanian Institute of Agrarian Economics, V. Kudirkos str. 18–2, 03105 Vilnius, Lithuania; justas.streimikis@gmail.com; 5 Faculty of Management and Finances, University of Economics and Human Science in Warsaw, Okopowa 59, 01-043 Warsaw, Poland

**Keywords:** external health costs, electricity generation, health indicators, dynamics, Baltic States

## Abstract

Implementation of strict policies for mitigating climate change has a direct impact on public health as far as the external health costs of electricity generation can be reduced, thanks to the reduction of emission of typical pollutants by switching to cleaner low carbon fuels and achieving energy efficiency improvements. Renewables have lower external health costs due to the lower life cycle emission of typical air pollutants linked to electricity generation, such as SO_2_, NOx, particulate matter, NH_3_, or NMVOC (Non-methane volatile organic compounds), which all appear to have serious negative effects on human health. Our case study performed in the Baltic States analyzed the dynamics of external health costs in parallel with the dynamics of the main health indicators in these countries: life expectancy at birth, mortality rates, healthy life years, self-perceived health, and illness indicators. We employed the data for external health costs retrieved from the CASES database, as well as the health statistics data compiled from the EUROSTAT database. The time range of the study was 2010–2018 due to the availability of consistent health indicators for the EU Member States. Our results show that the decrease of external health costs had a positive impact on the increase of the self-perceived good health and reduction of long-standing illness as well as the decrease of infant death rate. Our conclusions might be useful for other countries as well as for understanding the additional benefits of climate change mitigation policies and tracking their positive health impacts. The cooperation initiatives on clean energy and climate change mitigation between countries like One Belt One Road initiative by the Chinese government can also yield additional benefits linked to the public health improvements.

## 1. Introduction

Climate change mitigation policies aiming at greenhouse gas (GHG) reduction and implementation of Paris agreement provisions can provide considerable additional benefits linked to health improvement.

The European Union (EU) Member States (MS) implement strict policies to reduce GHG emissions and achieve significant progress in atmospheric emissions reduction linked to the electricity generation. The main typical pollutants related to the fossil fuel burning in electricity sector are SO_2_, NOx, particulate matter, NH_3_, and NMVOC. Although the main aims of climate change mitigation policies in the EU is reduction of GHG emissions and these reductions are mainly achieved by increasing energy efficiency and use of renewable energy sources in electricity generation, these actions also have a direct positive impact on emission reduction of those typical atmospheric pollutants.

Electricity generation from fossil fuels has significant negative public health impacts. The market failure to address environmental and human health damages in electricity generation made the fossil fuel-based electricity generation costs too low. Hence, policies and measures are required to correct the market failure and to ensure the level playing field for all electricity generation options. The pollution tax is a classic example of how such market failures can be corrected and how the external costs of electricity generation can be integrated into the price of electricity. In order to achieve the full integration of external costs of electricity generation via pollution taxes, the tax rates should be set equal to the sum of marginal external costs of each pollutant released into the atmosphere. However, there are no countries with such high pollution taxes for atmospheric pollutants emission, due to various political reasons [1]. Moreover, high pollution taxes are not accepted by companies and consumers. Nevertheless, there are other policies and measures dealing with the market failures, for example, climate change mitigation policies proposing various incentives for clean renewable fuels, financial initiatives in the form of capital subsidies or fiscal initiatives, and feed-in prices or feed-in-premiums etc.

Although there is a plethora of studies dealing with climate change mitigation policies and their impacts on GHG emission reduction, penetration of renewables and energy efficiency improvements, and overcoming various potential barriers for market uptake of clean energy generation technologies [2,3,4], the external health costs reduction based on the climate change policies and their positive health outcomes are not widely explored by researchers. Few local studies were developed trying to assess the external costs of electricity generation by employing damage estimates taken from other studies [5,6]; however, all these studies did not analyze the linkages between penetration of renewables and reduction of external health costs of electricity generation and did not address the linkages between external health costs reduction and improvement of health indicators.

The paper aims to address this gap and analyses the dynamics of external health costs of electricity generation due to implemented climate change mitigation policies and penetration of renewables in selected several EU Member States. The main input of this paper is finding answers to important questions about additional benefits of climate change mitigation measures linked to reduction of external health costs of electricity generation and improvement of health indicators. We are doing so by employing the primary assessment on the data of external health costs for selected countries and comparing the dynamics of state support for renewables and health indicators in selected countries. 

The rest of this paper is structured as follows: Section 2 presents literature review on the external health costs of electricity generation options. Section 3 introduces methods and data. Section 4 demonstrates the case study on the assessment of the evolution of external health costs of electricity generation and the trends in state support policies for renewables and relationship between external health costs and health indicators. Section 5 provides discussion of results. Finally, Section 6 concludes and lists main outcomes and policy implications.

## 2. Literature Review

Electricity generation and transportation represent the main sources of airborne emissions. The electricity generation costs are often of the same magnitude as private investments. However, they are often not fully accounted by the markets due to low pollution taxes [7]. The external costs should be fully assessed and integrated into the decision-making process. Nevertheless, the assessment of the external electricity generation may face great challenges due to the large amounts of data needed to carry out the analysis, as well as computation and monetization [8].

Several options for correcting market inefficiencies and integration of “externalities” linked to the electricity generation exist, like the use of fiscal instruments for integration of external costs and the promotion of renewable [9].

Generally, an effective control of the external costs of energy generation while pursuing energy consumption and further economic growth represents a complex problem. Therefore, the European Commission (EC) initiated many projects targeting the assessment and integration of energy externalities [10,11,12,13,14].

Electricity generation from fossil fuels leads to various pollutants emissions being released into the atmosphere. When the whole life cycle of fossil fuel is being considered, there are various pollutants emissions in the process of exploitation, transportation, and conversion of fossil fuel. The emission of the pollutants has negative impacts on air, water, soil, and human health. Damage to human health resulting from the poor air quality is regarded as the most serious effect of pollution linked to fossil fuel power generation cycle [15,16].

Power generation is the main source of the following so-called typical pollutants: Sulphur oxides and sulphates, nitrogen oxides and nitrates, VOC (volatile organic compounds), NH_3_, particulate matter and various metals, mercury, cadmium, and lead. Exposure to air pollution from power plants is related to various adverse human health effects. These negative effects are as follows: cardiovascular morbidity and mortality (e.g., strokes); and pulmonary morbidity and mortality (e.g., lung cancer and various respiratory diseases, such as asthma, which are especially dangerous for children) [17,18,19]. Particulate matter (PM), and especially the fine particulate matters with diameter less or equal to of 2.5 micrometers, or PM_2.5_, are the most dangerous pollutants from electricity generation process which have severe negative human health impacts [20,21,22].

With GHG emits to the earth’s atmosphere, the typical pollutants linked with electricity generation can cause many dangerous health effects. Operation of fossil fuel power plants caused a high negative health effect because the less advanced pollution abatement installations were applied in these plants [23].

With regard to the above, Epstein et al. [24] provided that the concentration-response function for PM_2.5_ established by Pope et al. [25] and applied in National Research Council [23] study and CASES [13] and also NEEDS [14] project are just “a low estimate for increases in mortality risk with the increases in PM_2.5_ exposure”. Epstein et al. [24] also indicated that in study by Schwartz et al. [26] the significantly higher estimate for PM_2.5_-related mortality was used comparing with the damage estimates derived by Pope et al. [17]. According to Epstein et al. [24], the application of dose-response function demonstrated in a study by Schwartz et al. [26] would cause three times higher damage estimates linked to particulate matter emission.

Machol and Rizk [27] also stated much higher external health costs in their study that dealt with comparison of the similar National Research Council [23] study results. In general, there are huge uncertainties in health damage estimates obtained in various studies. The main uncertainties are linked to the negative health effects of airborne pollutants and the values of a statistical life or a life year.

The most robust and recognized approach in assessing external costs of electricity generation is the Impact Pathway Approach developed in the EU as a part of several ExternE studies. ExtrenE approach is based on Multifaceted Dispersion Modelling, Industrial Source Complex Model (Gaussian plum model), and the regional Source Receptor matrices assessed by the integrated Eulerian Dispersion Model into Eco Sense Model. Industrial Source Complex Model is used for primary air pollutants chemical transport modelling on a local scale, i.e., 100 km × 100 km around each power plant [12,13,14].

There are regional external costs of electricity generation studies performed in various countries around the world. The main approach applied in these studies for the assessment of external costs of electricity generation is based on the impact pathway approach and ExternE methodology, including studies in Croatia [28], Bosnia and Hercegovina [29], Poland [30,31], and Greece [32]. In Iran [33], India [34], Cuba [35], South Africa [36], Mexico [37], Syria [38], and China [39,40], the simplified methodologies of the assessment of external costs of electricity generation were applied. There are several simplified methodologies: AirPacts model consisting of three different types of models, the Simple Uniform World Model (SUWM) requiring the least amount of data, the Robust Uniform World Model (RUWM), and QUERI (QUick Estimation of Respiratory health Impacts) models. The last two models were developed by integrating various correction factors to the SUWM model to improve its accuracy and reduce uncertainties. Other regional studies applied life cycle assessment (LCA) approach with various endpoint indicators frameworks for pollution impacts assessments. In a case study conducted in Brazil [41], the Life Cycle Impact (LCI) approach for assessing external costs of coal fired power generation was performed by applying two methods: the Eco-indicator 99 to evaluate five impacts categories and the IPCC GWP 100 years method to evaluate the global warming impact category. In Indonesian LCI study, the same approach was applied for assessment of external costs of coal supply chain [42]. Several LCA regional studies were conducted in Australia [43], China [44], and Caribbean islands [45].

The performed global comparison studies [46,47] used ExternE approach and average external costs data for electricity generation options. However, this is an important limitation due to application of European data for global scale.

The recent study [48] applied the Life Cycle Impact Assessment (LCIA) method based on endpoint modelling (LIME3) for G20 countries. The monetary health estimates of pollutants endpoints were performed based on performed Willingness to Pay assessments in G20 countries [49]. The detailed LCIA and LIME3 methodology is provided in [50]. The major limits of the LCIA study for G20 is the average monetary values for damage assessment applied for all G20 countries. This is an important limitation as far as the monetary damage estimates are linked with the price level and purchasing power parities (PPPs), which are very different for each G20 country.

## 3. Methods and Data

The assessment of the external health costs of electricity generation in the Baltic States is based on the data available from the CASES [13] project. The external health costs due to primary air pollutants (SO_2_, NOx and particulate matter) and their secondary emissions (nitrates, sulphates and ground level ozone) consequent to an average height of discharge were developed for 27 EU MS and other countries during CASES [13] project. The external health costs were assessed for the following atmospheric pollutants emission: NH_3_, NMVOC, NOx; SO_2_; PPMcoars, and PPM_25._ The receptor domain covered the whole of Europe in assessing the impacts to human health, and the EcoSence Model was applied.

Physical health impacts were calculated based on parameterized results of a Multifaceted Dispersion Modelling, the Industrial Source Complex Model (Gaussian plum model), and the regional Source Receptor matrices assessed by the integrated Eulerian Dispersion Model into the Eco Sense Model. The Industrial Source Complex Model was applied for primary air pollutants chemical transport modelling on a local scale, i.e., 100 km × 100 km around each power plant. The chemical transport modelling was used to assess the regional atmospheric dispersion and deposition of acidifying and eutrophying compounds ((S, N), ground level ozone (O_3_), and particulate matter (PM_10_, PM_2.5_)) originating from primary pollutants such as SO_2_, NOx, and particulates emission [13].

The main health impacts are associated with atmospheric emission of primary pollutants like particulates which is less than 10 or 2.5 microns in diameter, SO_2_, NO_2_, NMVOC, and secondary emissions represented by the sulfates and nitrates. The assessment of the health impacts of atmospheric pollution is usually conducted based on the so called “doze response functions” which link concentrations of different pollutants like particulate matter to the certain health outcomes assessed by physical units, such as loss of life years. These functions are derived from the epidemiological literature. Some of the key functions that have been identified due to the particulate maters and the related health impacts are shown in Table 1 that follows.

The impacts are assessed in terms of health endpoints such as number of years of life expectancy lost per microgram/per cubic meter a person is exposed to. Due to high uncertainties of their wide range of values, Table 1 just gave central values. The monetary values provided in Table 1 are obtained based on a range of methods [11]. One of the most recognized methods is assessment of willingness to pay for a reduction of such risks. The main negative health impacts of typical pollutants are summarized in Table 2.

External health costs were evaluated per unit of pollutants emission by heavy metals as well: Cadmium (Cd), Arsenic (As), Nickel (Ni), Lead (Pb), Mercury (Hg), Chromium, (Cr), Chromium IV (Cr-IV), and Formaldehyde and Dioxide. External health costs linked to metal emissions are not country-specific, and the same value for each EU member states can be considered. Furthermore, as variation of these damages with time is not assessed, the same result was applied to assess the external health costs in 2010, 2020 and 2030 in CASES [13].

All data on external health costs in EUR per unit of pollutants emitted and in EURct per kWh of electricity generated for specific energy carrier in Estonia, Latvia, and Lithuania was collected for this study were collected for Estonia, Latvia, Lithuania from project CASES data base [13]. In CASES project based on Eco Sense modelling results, the external cost for typical airborne pollutants were evaluated for each of the 27 EU MS. The values are based on the parameterized results of applied complex dispersion modeling. Results from CASES study were obtained for the following airborne emissions: NH_3_, NMVOC, NOx, PPMcoars, PPM_25_, and SO_2_. The selected receptor domain for the case studies covered all Europe. The health estimates were monetized in CASES study for each EU Member State based on Willingness to Pay studies, benefit transfer and are consistent with the ExternE approach developed for European Union [12].

The main health indicators for the Baltic States were collected from the EUROSTAT database: life expectancy in years, healthy life years in years, self-perceived good health in percent, infant death rate, and long-standing illness expressed in percent.

In order to find out the relationship between external costs and health indicators, the panel of three Baltic countries with 9 years of data from 2010 to 2018 was applied. The variables are: external health costs (EC) as the dependent variable, and the health indicators (independent variables) are life expectancy in years (LF), healthy life years in years (HL), self-perceived good health in percent (SPG), infant death rate (IDR), and long-standing illness in percent (LSI).

The resulting model can be expressed as follows:

(1) $$DEC_{j,t}=\sum_{j}^{m}\gamma_{j}+\sum_{t=1}^{T-1}\tau_{t}+\beta_{1}LF_{j,t}+\beta_{2}HL_{j,t}+\beta_{3}SPG_{j,t}+\beta_{4}IDR_{j,t}+\beta_{5}LSI_{j,t}+\in_{j,t}$$

where γs are the countries-specific dummies and m is the number of countries, τs is the yearly dummies, and *T* is the number of years.

The selection of fixed effect model is made by applying the Hausman Test [52]. This test is developed to explore the choice between fixed effects and random effects models. The null hypothesis (Ho) of no correlation, both OLS and GLS are consistent, but OLS is inefficient, against the alternative hypothesis (Ha) OLs is consistent whereas GLS is not. The advantage of the use of the fixed effect estimator is that it is consistent even when the estimators are correlated with the individual effect. The Hausman test uses the following test statistics:

(2) $$H={\left({\hat{\beta }}^{FE}-{\hat{\beta }}^{RE}\right)}^{\prime }{\left[Var\left({\hat{\beta }}^{FE}\right)-Var\left({\hat{\beta }}^{FE}\right)\right]}^{-1}\left({\hat{\beta }}^{FE}-{\hat{\beta }}^{RE}\right)\sim$$

If the value of the statistics is large, the difference between estimates is significant, so one can reject the Ho concluding the use of the fixed effect estimator. Alternatively, a small value for the Hausman statistic implies that the random effects estimator is more appropriate [52]. The result of the Hausman test revealed that we can use fixed effect model in order to estimate model (for detailed result see Annex).

Besides estimating full forms of the above model, we also attempted some other specifications by changing combinations of regressors as follows:

(3) $$DEC_{j,t}=\sum_{j}^{m}\gamma_{j}+\sum_{t=1}^{T-1}\tau_{t}+\beta_{1}HL_{j,t}+\beta_{2}SPG_{j,t}+\beta_{3}IDR_{j,t}+\beta_{4}LSI_{j,t}+\in_{j,t}$$

(4) $$DEC_{j,t}=\sum_{j}^{m}\gamma_{j}+\sum_{t=1}^{T-1}\tau_{t}+\beta_{1}HL_{j,t}+\beta_{2}SPG_{j,t}+\beta_{3}IDR_{j,t}+\in_{j,t}$$

In order to check the stationarity we applied the panel unit root test, Im, Pesaran and Shin W-stat (for detail result see the annexure); the p*vale of this test is 0.0029, which is less than 0.05 (95% confidence interval), so one may reject the Ho of unit root and conclude that the variables are stationary at levels or I(0).

## 4. Results of Case Study in the Baltic States

In the following sub-sections of this paper, the results of case study on evolution of external health costs of electricity generation and their impacts on health indicator in the Baltic States are provided, starting from analysis of external health costs of atmospheric emissions in the Baltic States.

### 4.1. External Health Costs of Atmospheric Emissions

External health costs of atmospheric pollution by electricity generation in the Baltic States retrieved from CASES are depicted in Table 3 that follows. The external health costs due to emission of formaldehyde, dioxide, and heavy metals in the Baltic States are also shown in Table 3. The external health costs due to the emission of formaldehyde, dioxide, and heavy metals are not site specific, and external costs of electricity generation technologies were evaluated by employing the same values for all EU member states, including the Baltic States CASES [13].

As specific electricity generation technologies have different life cycle atmospheric emissions, external health costs were assessed for the main electricity generation technologies in EU-27 MS during CASES project. The fossil fuel-based electricity generation technologies have significantly higher external health costs for the whole life cycle.

### 4.2. External Health Costs of Electricity Generation Technologies in Baltic States

**Figure 1:**
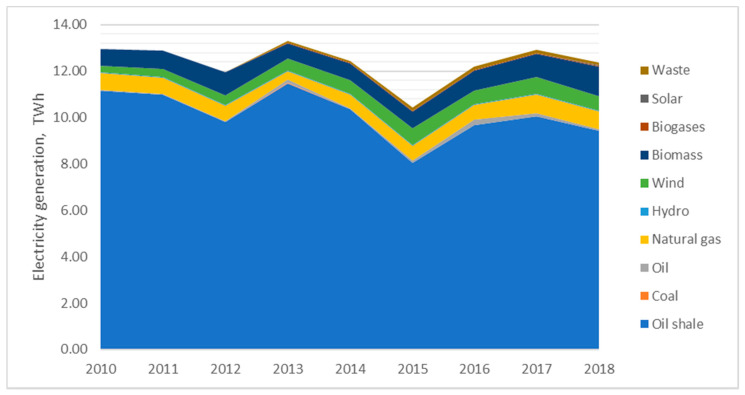
Dynamics of electricity generation structure in Estonia. Source: created by the authors based on the references [53].

External health costs of electricity generation depend on the structure of electricity generation in the Baltic States. These countries have very different electricity generation structure, like Estonia having local cheap oil shale resources which makes more than 70% in the structure of power generation even in 2018, though in 2010 the share of oil shale made 86% in electricity balance sheet of the country (Figure 1).

**Figure 2:**
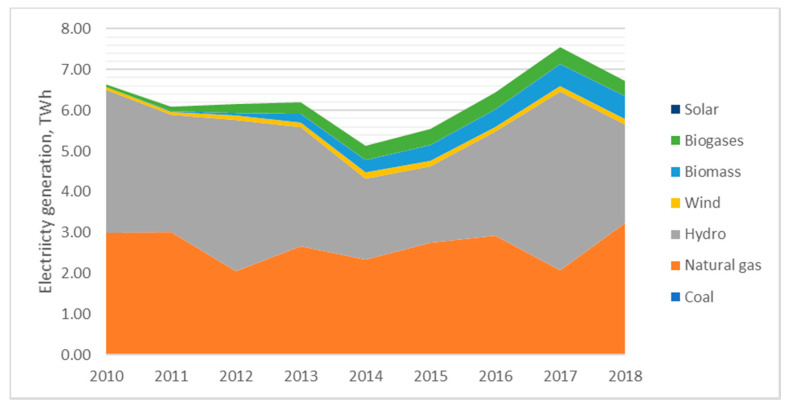
Dynamics of electricity generation structure in Latvia. Source: created by the authors based on the references [53].

Latvia distinguishes from other Baltic States with high share of hydro which decreased from 53% in 2010 to 36% in electricity balance sheet in 2018 (Figure 2).

**Figure 3:**
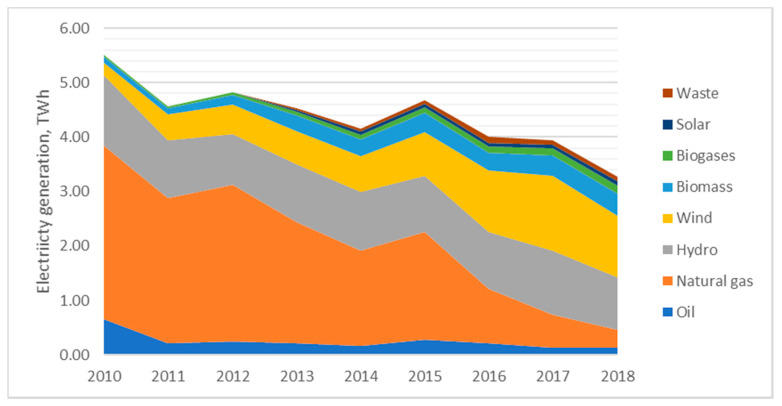
Dynamics of electricity generation structure in Lithuania. Source: created by the authors based on the references [53].

Lithuania distinguishes from its neighbors with very low electricity generation level; after the closure of Ignalina NPP in 2009, the country became a net energy importer. Even from 2010 to 2018, the domestic electricity generation declined from 5.5 GWh to 3.27 GWh. Currently, more than 70% of electricity production in the country comes from renewable energy resources (Figure 3).

Taking into account the difference of electricity generation structure, one can notice that Lithuania should have the lowest external health costs linked to power generation in recent years and Estonia should have the highest one as renewables can be characterized as energy carriers having the lowest life cycle external health costs of electricity generation in comparison with fossil fuels.

Following the methodology of CASES [13], the external life cycle health costs of the main electricity generation technologies were assessed for the Baltic States in EURcnt/kWh based on CASES database [13].

As CASES database on external costs of electricity generation was developed for EU MS for 2005–2010, 2020 and 2030, the external costs of energy generation technologies during 2010–2018 period for Baltic States were assessed by employing average values of the external health costs from CASES database during 2010–2020 period. The comparison of external life cycle health costs of electricity generation in the Baltic States in 2010–2020 average values are given in Table 4.

Based on the power generation structure of the Baltic States in 2010–2018, the total external health costs of electricity generation dynamics in the Baltic States are given in Table 5, Table 6 and Table 7.

During investigated period, the external costs have been reduced, however, the main input to high external health costs of electricity generation is provided by oil shale.

Comparing the external health costs of power generation in the three Baltic States it is obvious that Estonia has more than 10 times of higher external health costs of electricity generation comparing with Lithuania and Latvia. In addition, the level of electricity generation in Estonia was two times higher than in Latvia and almost four times higher than in Lithuania in 2018.

Lithuania in 2018 had the lowest external health costs of electricity generation in the Baltic States; however, in 2010, the lowest external health costs of electricity generation were in Latvia. Therefore, during 9 years period the external health costs of electricity generation in Lithuania more than halved. In Latvia the external costs of electricity generation increased slightly during investigated period due to increased consumption of natural gas and hydro by power generation. In Estonia the external health costs of electricity power decreased slightly (about 15%) from 2010 to 2018.

### 4.3. Dynamics of Health Indicators in Baltic States

Life expectancy is the core indicator of the public health in the country [54]. Other important health indicators are infant mortality (infant deaths against 1000 births); Standardized death rate per 100,000 inhabitants, Healthy life years at birth, Self-perceived good or very good health indicator, people having a long-standing illness older than 16 years old, etc. [54].

**Figure 4:**
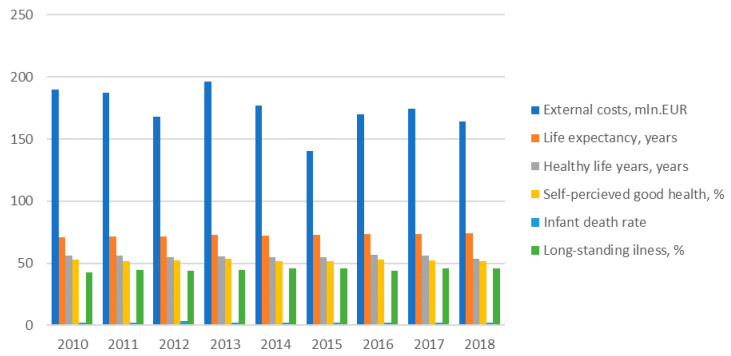
External health costs of power generation and health indicators development in Estonia. Source: own results.

**Figure 5:**
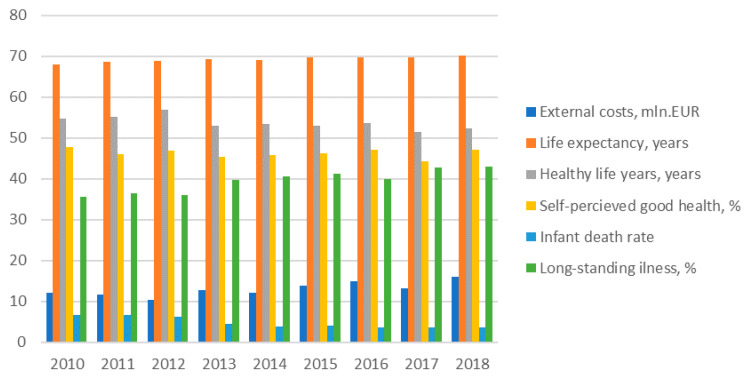
External health costs of power generation and health indicators development in Latvia. Source: own results.

**Figure 6:**
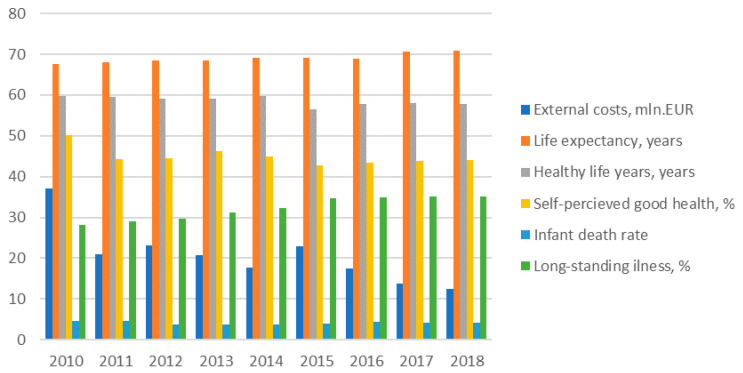
External health costs of power generation and health indicators development in Lithuania. Source: own results.

The dynamics of external health costs of electricity generation and other health indicators for three Baltic States are provided in Figure 4, Figure 5 and Figure 6 bellow.

The main health indicator—life expectancy at birth—has increased in Estonia though in general this country has very high external heath costs in comparison with other Baltic States. The positive trend of decreasing the external health costs of electricity generation might have positive impacts on health outcomes in the country. Other health indicators, such as healthy life years at birth and self-perceived good health, were slightly declining and the share of people having a long-standing illness was increasing in Estonia during investigated period. Therefore, though death rates including infant death were declining in the country during 2010–2018, the other health indicators showing negative trends in Estonia.

However, comparing the all health indicators in the Baltic States, it can be seen that Estonia distinguishes itself with the highest life expectancy and self-perceived very good health indicators though the best healthy life years at births and illness indicators are characteristics of Lithuania and at the same time there is the lowest self-perceived good health indicators in Lithuania. There is the highest and the lowest standardized death rate and infant death rates in Latvia and in Estonia respectively.

The life expectancy indicators were increasing in Latvia during the study period, though healthy life years were declining and illness indicators have increased during the same period. Standardized death rate and infant death rates were declining and self-perceived very good health remains quite stable during 2010–2018 period. Taking into account the slightly increased external health costs during this period and the relationship between them, the health indicators are thought to be ambiguous.

In Lithuania, during all investigated period, all death rates were decreasing and life expectancy was increasing; however, all other health indicators, i.e., health life years, self-perceived good health and illness indicators showed negative trends, and the situation is same in other Baltic States, showing that people tend to live longer but have poorer health.

### 4.4. Relationship between External Health Costs of Electricity Generation and Health Indicators

There are big differences in external health costs of electricity generation in Baltic States. Lithuania and Latvia have similar external health cost. However, Estonia distinguishes itself with ten times higher total external health costs of power generation due to local shale oil resources dominating in primary energy supply.

**Figure 7:**
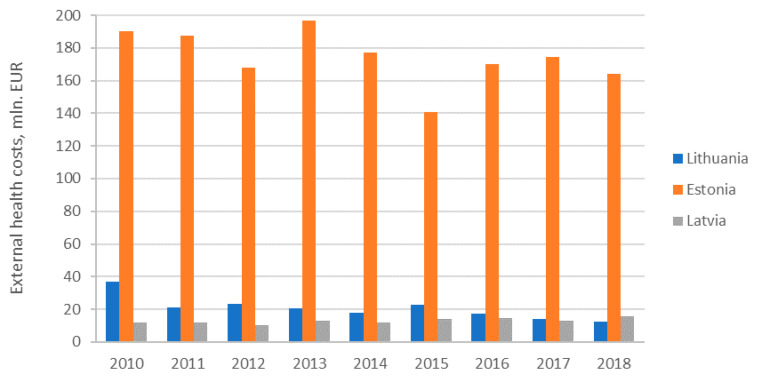
External health costs of power generation in the Baltic States. Source: Own results.

The dynamics of external health costs of electricity generation in the Baltic States is provided in Figure 7.

Analysis revealed that due to penetration of renewables external health costs were decreasing in all countries except Latvia (Figure 7). In Estonia, reduction of external health costs of electricity generation was accompanied by improvement of main health indicators. In Latvia, the increase of external health costs was accompanied by worsening of health indicators except life expectancy at birth rate and infant death rate. In Lithuania, the decrease of external health costs was accompanied by improving health indicators except long standing illness indicator which was worsening. In order to find out the relationship between external costs and health indicators, the panel of three Baltic countries was applied. The main variables are described in Methods and data section. An account of summary statistics of all selected variables is given in Table 8.

Table 8 shows that average external health cost of Latvia is minimum among the countries under study. Moreover, the highest external costs and highest variation are in Estonia among the other countries of the study. Furthermore, the average life expectancy is the minimum in Lithuania, with the minimum variation in Latvia. Healthy life years is the minimum in Latvia with lower variation also in Latvia. Self-perceived good health indicator has the minimum average in Lithuania and variation is the minimum in Estonia. Infant death rate on an average is the minimum in Estonia and the minimum standard deviation or variation is in Lithuania. The long-standing illness has minimum average in Lithuania whereas minimum variation in Estonia.

The pooled regression with bootstrap standard errors was also applied in the next step to analyze the relationship between external health cost (EC) and health indicators. For the analysis of the relationship between external health costs and health indicators, three estimated pooled regression models with bootstrapped standard error were developed in order to define statistically insignificant independent health variables and exclude them from pooled regression. In Model (1), all five independent health variables (LE, HL, SPG, IDR, and LSI) were included. In Model (2), four independent health variables (HL; SPG, IDR, and LSI) were left. In in the end, in Model (3), just three independent health variables (HL, SPG, and IDR) remained. In Table 9, results of three models are presented.

The results (Table 9) give evidence of negative relationship between external cost (EC) and all other health indicators except infant death rate (IDR). The significance of the sign of the relationship means that external costs has indirect relationship with infant death rate (IDR), whereas, for other indicators, the relationship has direct relationship. Among the three estimated pooled regression models with bootstrapped standard error (10000 repetitions), in the first model, out of five independent variables, two of them are not statistically significant. The insignificant indicators are life expectancy in years (LF) and long-standing illness in percent (LSI). Therefore, in the second model, the statistically insignificant indicator i.e., life expectancy in years (LF), was dropped and the result of estimated model of four independent variables reveals that, among these variables, the indicator long-standing illness in percent (LSI) is still statistically insignificant. The final model gives us the statistically significant indicators those having the relationship with external cost (EC). Among these three independent variables self-perceived good health in percent (SPG) and healthy life years in years (HL) indicators have negative statistically significant relationship with external costs at 95 percent confidence level, whereas the indicator infant death rate (IDR) has a positive and statistically significant relationship with external costs with same 95 percent confidence level.

### 4.5. Internalization of External Costs Baltic States

The main measures to internalize external health costs of electricity generation in energy sector are linked to promotion of renewables. The Baltic States have targets for renewable energy sources (RES) in 2020 and 2030 according to EU Climate and Energy package 20-20-20 and EU Climate and Energy Framework for 2030 [55]. Latvia has high share of RES in the gross final energy consumption due to its high hydro energy potential, which has led to the superior share of RES in its power generation structure. Lithuania is net energy importer, with an electricity import dependency above 70%. In Lithuania, the share of RES power generation is above 80%, but the share of RES in electricity consumption is above 18%, based on 2018 data.

There are these main types of support measures to support RES implemented in the Baltic States: price-based administratively regulation mechanisms (feed-in tariffs, providing guarantees to purchase renewable electricity at fixed price per kWh electricity, and feed-in premiums, which are adding premiums for renewables on the top of the market price of power), quantity-based or flexible market instruments (tradable green certificates with renewable quota obligations requiring the certain share of RES in power generation, tendering schemes or bidding systems providing opportunity for investors in RES projects to compete for supply contracts to build additional RES based electricity generation capacities), and other instruments (carbon taxes, tax allowances, and investment and financial incentives) [56].

The main policies and measures targeting RES development in power sector are financial instruments and administratively set pricing schemes. In Table 10 the administratively set pricing mechanisms to support renewables implemented in the Baltic States are presented.

The feed-in premium tariffs are applied just in Lithuania and Estonia (Table 10). The existing feed-tariff in Latvia were suspended until 1 January 2020 due to problems linked to the lack of transparency and corruption risks; however, up to now there are no other system adopted. The rates of tariffs for all technologies are the same in Estonia; however, in Lithuania the system is much more complicated and different feed-in price premiums are applied for specific technologies. The solar energy distinguishes with the highest premiums, which are more than two times higher than that for hydro and wind. One can notice the high premiums for biogas in Lithuania. It is worth mention that these premium tariffs are not linked with external costs of renewable technologies or avoided external costs of switching from fossil fuels to these technologies.

The major revisions in the legislation linked to RES support have been implemented in latest years in all Baltic States. An auction-based system to promote RES development has been implemented since 2018 in Estonia and Lithuania. The public tenders based on reverse auction principles were adopted to achieve the national RES objectives in electricity consumption up to 2020.

The dynamics of public support for RES in the Baltic States is provided in Table 11.

Though Latvia distinguishes itself with the highest support rates to renewable electricity, the external health costs in this country are more than ten times lower than that in Estonia and the share of RES in electricity generation has not increased sharply, as in the case of Lithuania (Table 11). The support to renewable electricity per kWh is the lowest in Estonia and, taking into account the current high external heath costs of electricity generation, support for renewables should be increased. Lithuania has a twice higher state support for renewables, compared to Estonia, and a twice lower support, compared to Latvia.

The positive trend of the increasing share of renewables in power generation of Lithuania during 2010–2018 period and halving of external health costs of electricity generation provides a positive example that it is possible to achieve significant progress in penetration of renewables and reducing external costs linked to power generation with moderate state support to renewables.

## 5. Discussion

The aim of the study was to investigate the evolution of external health costs of electricity generated in the selected three EU Member States having similar social-economic situations and similar policies. The countries in question implemented similar policies and measures to promote renewables and to achieve 100% of renewables in electricity generation by 2050.

The increase of the share of renewables in electricity generation provides for the decrease of external health costs as renewables distinguish with the lower life cycle external health costs comparing to renewables. Therefore, fast penetration of renewables, together with climate change mitigation benefits, should provide for extra benefits linked to improvement on public health.

Though there are several studies dealing with assessment of external costs of electricity generation in EU Members States such as Croatia [28], Bosnia and Hercegovina [29], Poland [30,31], and Greece [32] etc., these studies did not analyze the linkages between penetration of renewables and reduction of external health costs. The study by Ortega-Izquierdo and del Rio [59] and Ortega et al. [60] analyzed the benefits and costs of renewable electricity in EU member states and have found that benefits of renewable energy penetration due to avoided GHG emissions and fuel savings were higher than support provided for internalization of external benefits of renewable energy sources.

The current study analyzed (not avoided) external costs, and external health costs dynamics, due to penetration of renewables, and tried to assess additional public health benefits for selected three EU Member States in the Baltic region. The analysis of evolution of external health costs of electricity generation in the Baltic States revealed different trends in the three countries, though all these countries pursue the same policies to promote renewables and achieve carbon free economy in 2050 and Paris commitments set by European Union. In addition, it is necessary to stress that there are very large differences in external health costs of electricity generation in the Baltic States. Lithuania and Latvia have a similar external health cost; however, Estonia distinguishes itself with ten times higher total external health costs of power generation due to local shale oil resources dominating in primary energy supply of the country. With increase of the share or renewables and during transition towards low carbon future, external health costs reduction and improvement of health indicators is expected; however, due to penetration of renewables external, health costs were decreasing only in Estonia and Lithuania. In addition, Latvia distinguishes itself with the highest support rates to renewable electricity; however, the share of RES in electricity generation has not increased sharply in Latvia like in other Baltic States. Though the support to renewable electricity per kWh is the lowest in Estonia, the country has achieved good results of penetration of renewables and external health costs reduction.

Lithuania can also be used as a good example of a sharp increase of the share of renewables in power generation during 2010–2018 period and halving of external health costs of electricity generation. Significant progress in penetration of renewables and reducing external costs linked to power generation was achieved in Lithuania with moderate state support to renewables.

In Estonia, the reduction of external health costs of electricity generation was accompanied by improvement of main health indicators. In Latvia, the increase of external health costs was accompanied by worsening of health indicators, except life expectancy at birth rate and infant death rate. In Lithuania, the decrease of external health costs was accompanied by improving health indicators, except long standing illness indicator which was worsening. However, the performed analysis of the relationship between external costs and health indicators in panel of three Baltic countries revealed that only few indicators, including self-perceived good health and healthy life years, have a negative statistically significant relationship with external health costs at 95 percent confidence level. The infant death rate also has positive statistically significant relationship with external health costs with the same 95 percent confidence level; however, for other health indicators, such as life expectancy at birth and long-standing illness, a statistically insignificant relationship with external health costs was obtained.

## 6. Conclusions

Overall, the performed assessment of the external health costs of electricity generation trends in Baltic States during the 2010–2018 period showed that Estonia has more than 10 times higher external health costs of electricity generation comparing with Lithuania and Latvia. In addition, the level of electricity generation in Estonia was two times higher than that in Latvia and almost four times higher than that in Lithuania in 2018. This is because Lithuania has more than 80% of electricity generated by renewables and Estonia has almost 80% of dirty oil shale in its current electricity generation structure and Latvia has 50% of power generation sheet from natural gas.

The analysis on the trends of main health indicators in the Baltic States revealed that, throughout all of the investigated period, death rate indicators were decreasing and life expectancy indicators were increasing in all Baltic States; however, all other health indicators, i.e., health life years, self-perceived good health, and illness indicators, were exhibiting negative trends and providing that people tend to live longer but have a poorer health.

The statistical data analysis showed that self-perceived good health and healthy life years have negative statistically significant relationship, with external health costs at 95 percent confidence level. The infant death rate has positive statistically significant relationship with external health costs, with the same 95 percent confidence level; however, for other health indicators, such as life expectancy at birth and long standing illness, the statistically insignificant relationship with external health costs was obtained.

The study has limitations due to limited data availability (three countries and nine observations per country). The other limitations are related with the fact the dynamics of health indicators in the country are also linked with other important health drivers (quality of health care, education, alcohol and tobacco consumption, food consumption, lifestyle etc.) and age of inhabitants; therefore, the relationship between external health costs and other main statistical data on health indicators should be treated with caution, as the external health costs analyzed in this study are mainly related to atmospheric pollution. Therefore, future research is necessary and panel data analysis for all European Union Member States should be applied in in future research.

Taking into consideration the performed analysis, the following policy implications were developed: fossil fuels have higher life cycle external health costs compared to renewables, and internalization of these external costs are necessary to create the level playing ground for renewables. Therefore, renewable electricity generation technologies should be supported by internalizing the avoided external costs of renewables in comparison with fossil fuels.

The analysis of policies to promote renewable energy sources in the Baltic States provided that Latvia distinguishes itself with the highest state support rates for renewable electricity, though the external health costs in this country are more than ten times lower than that in Estonia and only slightly higher than that in Lithuania. Lithuania has a twice higher state support for renewables compared to Estonia and a twice lower support compared to Latvia. One can see that there is the lowest state support for renewable electricity per kWh in Estonia, which distinguishes itself with the highest external health costs of electricity generation. Therefore, the support for renewables should be increased in Estonia.

Even though in Estonia and Latvia, feed-in premiums were set equal for all technologies seeking to ensure that energy is produced using the most cost-effective technologies and guarantee the technology-neutral support schemes, this approach desires criticism as renewables have different external costs and the internalization of these costs requires technology specific support.

All in all, it appears that the future energy and climate policies should also take into account the difference of external costs of electricity generation technologies and provide supports for these technologies by taking into account external health costs of electricity generation.

## Figures and Tables

**Table 1:** Health impacts and their monetary values for PM linked to electricity generation from fossil fuels.

Airborne Pollutants	Physical Impact	Monetization of Physical Impacts, EUR
PM diameter < 2.5 microns		
Reduction of Life Expectancy (Years)	6.51 × 10^−4^	40,000
Restricted Activity Days	3.69 × 10^−2^	38
Days of Work Lost	1.39 × 10^−2^	295
Restricted Activity Days	9.59 × 10^−3^	130
PM diameter < 10 microns		
Infant’s increased Risk of Mortality	6.84 × 10^−8^	3,000,000
Chronic Bronchitis (new cases)	1.86 × 10^−3^	200,000
Respiratory Hospital Admissions	7.03 × l0^−6^	2000
Cardiological Hospital Admissions	4.36 × 10^−6^	2000
Adult’s Lower Respiratory Symptoms	3.24 × 10^−2^	38
Children’s Lower Respiratory Symptoms	2.08 × 10^−2^	38

Source: created by authors based on the reference [51].

**Table 2:** Health impacts of atmospheric emissions linked to electricity generation.

Primary Pollutants	Secondary Pollutants	Impacts
Particulate matter (PM_10_, PM_2.5_)		Mortality and Morbidity (congestive heart failure, chronic bronchitis, chronic cough of children, lower respiratory symptoms, asthma etc.)
**SO_2_**		Mortality and Morbidity (hospitalization, asthma, sick leave, restricted activity days)
**SO_2_**	Sulfates	The same like for Particulate Matters
**NO_2_**		Morbidity
**NO_2_**	Nitrates	The same like for Particulate Matters
**NO_2_ + VOC**	Ozone	Mortality and Morbidity (respiratory hospital admissions, restricted activity days, asthma etc.)

Source: created by the authors based on the reference [51].

**Table 3:** External health costs of atmospheric emissions of primary pollutants in the Baltic States, 2005EUR/t.

Pollutants	Estonia	Lithuania	Latvia
**Human Health Impacts of Classical Pollutants**			
NH_3_	3323	2371	2901
NMVOC	26	56	35
NOx	2064	4653	3294
PPM co	190	397	342
PPM_25_	7279	11,169	9371
SO_2_	3653	5017	4343
**Human Health Impacts of Metals**			
Cd	46,200	46,200	46,200
As	94,700	94,700	94,700
Ni	4700	4700	4700
Pb	710,600	710,600	710,600
Hg	10,421,800	10,421,800	10,421,800
Cr	37,300	37,300	37,300
Cr-IV	284,200	284,200	284,200
Formaldehyde	236,900	236,900	236,900
Dioxine	4.40 × 10^13^	4.40 × 10^13^	4.40 × 10^13^

Source: created by the authors based on the reference [13].

**Table 4:** External health costs of electricity generation in Baltic States in 2010–2020 average values, EURct/kWh.

	Estonia	Latvia	Lithuania
Oil shale	1.66702	-	-
Coal	0.2942	0.4629	0.4629
Oil	1.667	2.385	3.1645
Gas	0.2397	0.3341	0.4361
Hydro	0.0609	0.0417	0.0989
Wind	0.0632	0.192	0.0753
Biomass	0.1782	0.2355	0.7527
Biogas	0.613	0.6826	0.7527
Solar	0.1612	0.192	0.2229
Waste	0.5635	0.72	0.8824
Nuclear	0.4144	0.4254	0.4367

Source: created by the authors based on the reference [13].

**Table 5:** Evolution of external health costs of power generation in Estonia during 2010–2018, million (mln.) EUR.

Fuels	2010	2011	2012	2013	2014	2015	2016	2017	2018
Oil shale	186.21	183.21	163.37	191.54	172.70	134.03	161.20	167.37	157.20
Coal	0.00	0.00	0.00	0.03	0.03	0.00	0.00	0.06	0.00
Oil	0.67	0.67	1.00	2.17	0.67	2.17	4.33	2.00	1.33
Natural gas	1.70	1.63	1.51	0.89	1.39	1.49	1.46	1.97	1.82
Hydro	0.02	0.02	0.02	0.02	0.02	0.02	0.02	0.02	0.01
Wind	0.18	0.23	0.27	0.33	0.38	0.46	0.37	0.46	0.40
Biomass	1.30	1.37	1.76	1.16	1.30	1.27	1.50	1.78	2.26
Biogases	0.06	0.12	0.12	0.12	0.18	0.31	0.31	0.25	0.25
Solar	0.00	0.00	0.00	0.00	0.00	0.00	0.02	0.02	0.05
Waste	0.00	0.00	0.00	0.34	0.39	0.73	0.73	0.79	0.56
Total	190.13	187.25	168.06	196.60	177.07	140.46	169.95	174.70	163.89

Source: created by the authors based on the references [13,53].

**Table 6:** Evolution of external health costs of power generation in Latvia during 2010–2018, mln. EUR.

Fuels	2010	2011	2012	2013	2014	2015	2016	2017	2018
Coal	0.00	0.00	0.00	0.00	0.00	0.00	0.00	0.00	0.05
Natural gas	9.99	10.06	6.88	8.92	7.82	9.22	9.82	6.92	10.76
Hydro	1.47	1.21	1.55	1.21	0.83	0.78	1.06	1.83	1.01
Wind	0.10	0.13	0.21	0.23	0.27	0.29	0.25	0.29	0.23
Biomass	0.02	0.02	0.14	0.49	0.75	0.89	1.01	1.25	1.34
Biogasses	0.41	0.75	1.50	1.98	2.39	2.66	2.73	2.80	2.53
Solar	0.00	0.00	0.00	0.00	0.00	0.00	0.00	0.00	0.00
Total	11.99	11.69	10.28	12.84	12.06	13.84	14.87	13.08	15.92

Source: created by the authors based on the references [13,53].

**Table 7:** Evolution of external health costs of electricity generation in Lithuania during 2010–2018, mln. EUR.

Fuels	2010	2011	2012	2013	2014	2015	2016	2017	2018
Oil	20.57	6.65	7.59	6.65	5.06	8.86	6.96	4.43	4.11
Natural gas	13.91	11.64	12.56	9.68	7.63	8.63	4.32	2.62	1.44
Hydro	1.29	1.05	0.93	1.06	1.08	1.01	1.03	1.17	0.95
Wind	0.17	0.35	0.41	0.45	0.48	0.61	0.86	1.02	0.86
Biomass	0.90	0.90	1.35	2.26	2.41	2.71	2.41	2.86	3.01
Biogases	0.23	0.30	0.30	0.45	0.60	0.68	0.90	0.98	1.05
Waste	0.00	0.00	0.00	0.26	0.35	0.53	0.88	0.71	0.71
Solar	0.00	0.00	0.00	0.00	0.00	0.00	0.09	0.09	0.26
Total	37.06	20.90	23.15	20.81	17.62	23.03	17.45	13.87	12.40

Source: created by the authors based on the references [13,53].

**Table 8:** Summary statistics of variables (in log form) from 2010 to 2018.

	External Health Costs (EC)	Life Expectancy (LE)	Healthy Life Years (HL)	Self-Perceived Good Health	Infant Death Rate (IDR)	Long-Standing Illness (LSI)
	**Latvia**					
Mean	2.553	4.238	3.983	3.834	1.526	3.673
Standard Deviation	0.133	0.010	0.030	0.023	0.265	0.073
Maximum	2.768	4.250	4.040	3.865	1.887	3.761
Minimum	2.330	4.218	3.940	3.789	1.281	3.572
Observations	9	9	9	9	9	9
	**Lithuania**					
Mean	2.983	4.235	4.071	3.804	1.423	3.470
Standard Deviation	0.320	0.016	0.019	0.048	0.088	0.090
Maximum	3.613	4.261	4.091	3.916	1.548	3.558
Minimum	2.518	4.214	4.034	3.757	1.303	3.336
Observations	9	9	9	9	9	9
	**Estonia**					
Mean	5.156	4.285	4.017	3.958	0.907	3.801
Standard Deviation	0.100	0.016	0.015	0.012	0.138	0.028
Maximum	5.281	4.304	4.010	3.980	1.250	3.833
Minimum	4.945	4.261	3.987	3.942	0.761	3.752
Observations	9	9	9	9	9	9

Source: Own results.

**Table 9:** Pooled regression results with Bootstrap Standard Errors (dependent variable is EC and all variables are in log form).

Variables	Model 1	Model 2	Model 3
EC	−67.952	−66.305	−47.966
	(−2.663)	(−3.815)	(−5.092)
LE	0.6888		
	(0.0960)		
HL	7.991 *	8.219 **	4.374 **
	(1.901)	(2.495)	(2.701)
SPG	8.423 **	8.420 **	9.337 **
	(4.537)	(4.855)	(5.634)
IDR	−1.356	−1.371 **	−1.684 **
	(−2.791)	(−3.160)	(−5.261)
LSI	1.540	1.667	
	(0.809)	(1.284)	
Adjusted R^2^	0.910	0.920	0.920
Standard Error of Regression	0.340	0.340	0.340
Number of Observations	27	27	27

Notes: t-statistics are reported in parenthesis; * Significant at 10 percent level; ** Significant at 5% level. Source: own results.

**Table 10:** Feed-in premiums in the Baltic States in 2020.

Renewables	Lithuania	Estonia
Wind Power Plants (PP)	Installed capacity < 10 kW: 5.2 €c/kWh Installed capacity > 10 kW < 350 kW: 5.0 €c/kWh Installed capacity > 350 kW: 4.1 €c/kWh	5.4 €c/kWh
Solar installations	Building-integrated solar installations: Installed capacity < 10 kW: 13.6 €c/kWh Installed capacity > 10 kW <100 kW: 12.4 €c/kWh Installed capacity >100 kW < 350kW: 11.5€c/kWh Installed capacity > 350 kW: 12.2€c/kWh Solar installations not integrated in buildings: Installed capacity < 10 kW: 16.9 €c/kWh Installed capacity > 10 kW < 100 kW: 15.2 €c/kWh Installed capacity >100 kW < 350kWh: 14.1 €c/kWh Installed capacity > 350 kWh: 14.8 €c/kWh	5.4€c/kWh
Geothermal PP energy	-	5.4€c/kWh
Biogas PP	PP using landfill gas: Installed capacity < 10 kW: 11.1 €c/kWh Installed capacity >10 kW < 500 kW: 10.6 €c/kWh Installed capacity > 500 kW: 8.6 €c/kWh PP using biogas derived from anaerobic digestion: Installed capacity < 10 kW: 13.4 €c/kWh Installed capacity >10 kW < 500 kW: 12.2 €c/kWh Installed capacity >500 kW < 1000 kW: 11.6 €c/kWh Installed capacity >1000 kW < 2000 kW: 11.0 €c/kWh Installed capacity > 2000 kW: 10.7 €c/kW	5.4€c/kWh
Hydro PP	Installed capacity < 10 kW:5.9€c/kWh Installed capacity > 10 kW < 1000 kW: 5.3€c/kWh Installed capacity > 1000 kW: 3. €c/kWh	5.4€c/kWh
Biomass PP	New PP using biomass: Installed capacity < 10 kW: 6.6 €c/kWh; Installed capacity > 10 kW < 5000 kW: 5.7€c/kWh Installed capacity > 5000 kW: 5.1 €c/kWh Reconstructed PP using biomass: Installed capacity < 10 kW: 4.6 €c/kWh Installed capacity > 10 kW < 5000 kW: 4.0 €c/kWh Installed capacity > 5000 kW: 3.5 €c/kWh	5.4€c/kWh

Source: created by the authors based on the reference [57].

**Table 11:** Evolution of public support to RES in the Baltic States during 2012–2017, EUR/kWh.

Electricity Generation Technologies	2010	2011	2012	2013	2014	2015	2016	2017
**Estonia**								
Solar	-	-	-	-	16.09	22.62	20.64	20.50
Hydro	51.85	51.61	14.50	10.56	16.09	22.62	20.64	20.50
Wind	53.48	53.68	14.50	10.56	16.09	22.62	20.64	20.50
Biomass	53.64	53.68	14.50	10.56	16.09	22.62	20.64	20.50
Total	53.55	53.66	14.50	10.56	16.09	22.62	20.64	20.50
**Latvia**								
Solar					-	-	-	-
Hydro					130.03	138.42	143.32	137.41
Wind					5.17	67.28	70.47	72.71
Biomass					129.42	138.42	143.32	137.41
Total					120.22	117.61	104.85	117.44
**Lithuania**								
Solar			367.82	191.90	119.21	116.68	322.95	326.48
Hydro			29.45	25.97	24.67	21.10	33.51	36.99
Wind			52.62	44.80	31.00	22.60	46.10	45.00
Biomass			88.90	69.60	47.76	24.58	51.40	55.48
Total			59.33	56.18	39.64	28.64	58.74	56.42

Source: created by the authors based on the reference [58].
